# Uric acid and uric acid to creatinine ratio in the assessment of chronic obstructive pulmonary disease: Potential biomarkers in multicomponent models comprising IL-1beta

**DOI:** 10.1371/journal.pone.0234363

**Published:** 2020-06-05

**Authors:** Lada Rumora, Iva Hlapčić, Sanja Popović-Grle, Ivana Rako, Dunja Rogić, Ivana Čepelak

**Affiliations:** 1 Department of Medical Biochemistry and Hematology, Faculty of Pharmacy and Biochemistry, University of Zagreb, Zagreb, Croatia; 2 University Hospital Centre Zagreb, Clinical Department for Lung Diseases Jordanovac, Zagreb, Croatia; 3 School of Medicine, University of Zagreb, Zagreb, Croatia; 4 University Hospital Centre Zagreb, Clinical Institute of Laboratory Diagnostics, Zagreb, Croatia; National and Kapodistrian University of Athens, GREECE

## Abstract

Chronic obstructive pulmonary disease (COPD) is a complex and heterogeneous disease, with oxidative stress and inflammation implicated in its development. Uric acid (UA) could exert anti-oxidative, pro-oxidative or pro-inflammatory effects, depending on the specific context. It was recently shown that soluble UA, and not just its crystals, could activate the nucleotide-binding oligomerization domain-like receptor family pyrin domain-containing 3 (NLRP3) inflammasome, leading to interleukin (IL)-1β secretion. We aimed to assess the differences in blood levels of UA and its ratio with creatinine (UCR) between COPD patients and healthy subjects, as well as their association with disease severity, smoking status, common COPD comorbidities and therapy regimes. The diagnostic characteristics of UA and UCR were also explored. This study included 109 stable COPD patients and 95 controls and measured white blood cells (WBC), C-reactive protein (CRP), fibrinogen (Fbg), IL-1β, creatinine (CREAT) and UA. All of the parameters were increased in COPD patients, except for CREAT. UA and UCR were positively associated with WBC, CRP and IL-1β. COPD smokers had lower UA and UCR values. Common COPD therapy did not affect UA or UCR, while patients with cardiovascular diseases (CVD) had higher UA, but not UCR, levels. Patients with higher UCR values showed worse disease-related outcomes (lung function, symptoms, quality of life, history of exacerbations, BODCAT and BODEx). Also, UCR differentiated patients with different severity of airflow limitation as well as symptoms and exacerbations. The great individual predictive potential of UCR and IL-1β was observed with their odds ratios (OR) being 2.09 and 5.53, respectively. Multiparameter models of UA and UCR that included IL-1β were able to correctly classify 86% and 90% of cases, respectively. We suggest that UA might be a useful biomarker when combined with IL-1β, while UCR might be even more informative and useful in overall COPD assessments.

## Introduction

Despite continuous and intensive effort from the side of health care providers, scientists and pharmaceutical industry, numbers regarding chronic obstructive pulmonary disease (COPD) outcomes associated with quality of life, morbidity and mortality are not improving and more than 3 million people die from COPD each year. In fact, COPD is still an under-recognized and under-diagnosed disease, so the actual mortality rate is probably much higher. It was predicted that in 2040 COPD will become the fourth leading cause of death [1]. Morbidity due to COPD is also increasing and may be affected by other concomitant chronic conditions like cardiovascular diseases (CVD) and metabolic syndrome (MS), while in COPD patients the development of comorbidities may be seen already at an earlier age [2]. Therefore, studies in the field of COPD are of the utmost importance for public health.

The pathogenesis of COPD is very complex and heterogeneous, and both oxidative stress and chronic low-grade inflammation are among the mechanisms proposed for COPD development. These disturbances are present not just locally in the respiratory system, but also throughout the organism, and systemic inflammation is recognized as one of the possible endotypes of COPD [3,4].

In the never-ending search for diagnostic and/or prognostic biomarkers in COPD assessment, some authors have found higher concentrations of uric acid (UA) in COPD patients in comparison to healthy subjects, and suggested that increased UA production could be a consequence of greater purine catabolism secondary to tissue hypoxia present especially in more severe disease stages [5–8]. Elevated UA levels might interfere with redox and inflammatory processes, which are altered in COPD. The molecular mechanisms of UA action are complex and could have opposing roles, e.g. anti-oxidative and pro-oxidative, with the prevailing one depending on specific contexts [9–11]. In addition, it has been suggested that UA may exert an inflammation-stimulatory effect, as soluble UA induced C-reactive protein (CRP) expression [12] in experimental studies, as well as the production of tumor necrosis factor α (TNFα), interleukin (IL)-6 and also IL-1β [13]. Indeed, recent data confirmed that not just crystals of monosodium urate (MSU) but also its soluble form possess danger-associated molecular pattern (DAMP)-assigned characteristics and can activate the nucleotide-binding oligomerization domain-like receptor family pyrin domain-containing the 3 (NLRP3) inflammasome [14–20]. Upon its activation, the autoactivation of caspase-1 is triggered, thus promoting the maturation and secretion of IL-1β and IL-18 [21,22]. There has been growing evidence implicating NLRP3 inflammasome activation in the inflammation observed in COPD [23,24].

In humans, UA is the final product of the purine nucleotides catabolism that involves several enzymes, with xanthine dehydrogenase/oxidase (XO) being a rate-limiting one [25]. UA is mostly disposed of by the kidney (about 70%) and therefore kidneys play a critical role in maintaining UA homeostasis and its plasma concentration. Impaired renal excretion leads to hyperuricemia (>339 μmol/L in premenopausal women and >416 μmol/L in men and postmenopausal women) [13,25]. Because excretion of UA is highly dependent on kidney function, the assessment of its corrective ratio with creatinine i.e. uric acid to creatinine ratio (UCR) is also important. It was reported that higher serum UA concentrations are associated not just with gout and renal diseases, but also with CVD and MD among others [26–28].

It has also been suggested that the delicate balance of dichotomous DAMP and antioxidant functions of UA may be affected by its altered levels, which may contribute to the development of chronic diseases, and COPD might be one such disease. We have previously found that patients with stable COPD have higher concentrations of ceruloplasmin and malondialdehyde, and lower concentrations of albumin, transferrin and thiols, thereby confirming systemic redox imbalance [29]. In addition, we also observed higher levels of extracellular ATP, CRP, fibrinogen (Fbg), white blood cells (WBC) and platelets counts that confirmed systemic inflammation [30,31]. In this study, our aim was to assess the levels and diagnostic potential of serum UA and UCR in COPD patients during the stable phase of the disease. Our primary goal was to investigate the association of UA and UCR with COPD disease severity, smoking history and the most common comorbidities and COPD therapy regimes. Our secondary goal was to propose the best multiparameter models with UA or UCR that might differentiate healthy and COPD individuals.

## Participants and methods

### Participants

The COPD patients (n = 109) and healthy subjects were matched by both sex and age (n = 95) and voluntarily participated in this study, signing an informed consent. The study was approved by the Ethics Committee of the University Hospital Centre Zagreb (Zagreb, Croatia) and by the Ethics Committee for Experimentation of the Faculty of Pharmacy and Biochemistry, University of Zagreb (Zagreb, Croatia). All patients were in the stable phase of their disease with no exacerbations during the last three months, without any changes in their therapy regime and without infections in the lower respiratory tract. They were recruited at the Clinical Department for Lung Diseases Jordanovac, University Hospital Centre Zagreb, during 2017 and 2018 by a pulmonology specialist who diagnosed the disease based on anamnestic and clinical data, current symptoms and value of spirometric ratio forced expiratory volume in one second (FEV_1_)/ forced vital capacity (FVC) < 0.70, according to the Global Initiative for Chronic Obstructive Lung Disease (GOLD) recommendations [2]. The COPD patients were subdivided by the severity of airflow limitation assessed by FEV_1_ as follows: FEV_1_ ≥ 80% represent GOLD 1 stage (n = 0), 50% ≤ FEV_1_ < 80% represent GOLD 2 stage (n = 39), 30% ≤ FEV_1_ < 50% represent GOLD 3 stage (n = 36), and FEV_1_ < 30% represent GOLD 4 stage (n = 34). In addition, according to the severity of symptoms and history of exacerbations, patients were classified into GOLD A group (n = 14), GOLD B group (n = 63), GOLD C group (n = 0) and GOLD D group (n = 32), with the COPD Assessment Test (CAT) score used for the symptoms assessment. Regarding comorbidities, patients were subdivided according to the presence of CVD and MS, as those are the most frequent COPD comorbidities. In this study, the term CVD encompassed arterial hypertension, coronary artery disease, congestive heart failure, and atrial fibrillation, while the term MS encompassed diabetes mellitus, osteoporosis and hyperlipidaemia. Patient comorbidities were determined after a detailed medical history, physical examination, and study of their medical record. Regarding therapy, COPD patients were subdivided by the most common COPD therapy regimes recommended by GOLD as follows: patients in group 1 (n = 20) received monotherapy of long-acting bronchodilator (long-acting β_2_-agonists (LABAs) or long-acting muscarinic antagonists (LAMAs) with or without short-acting bronchodilator (short-acting β_2_-agonists (SABAs) or short-acting muscarinic antagonists (SAMAs), group 2 (n = 32) received dual long-acting bronchodilators LABA and LAMA, group 3 (n = 20) received a combination of long-acting bronchodilators with inhaled corticosteroids (ICS), and group 4 (n = 37) received triple therapy with added LAMA.

The health status of the control group was evaluated by anamnestic and spirometric data. The inclusion and exclusion criteria were the same for both controls and patients, meaning that they all had to be older than 40 years, could not have any lung diseases (except COPD for COPD patients), could not have any inflammatory diseases, acute infections, gout, kidney diseases, liver diseases, malignant diseases, transplantations and other specific or non-specific ongoing inflammations. We did not include individuals who used drugs that can affect uric acid metabolism and cause hyperuricemia. Healthy subjects and patients were also subdivided according to their self-reported smoking history into healthy non-smokers (n = 48), healthy current smokers (n = 47), COPD non-smokers (n = 5), COPD ex-smokers (n = 75) and COPD current smokers (n = 29) groups. St. George’s respiratory questionnaire for COPD patients (SGRQ-C), modified Medical Research Council (mMRC) Dyspnea Scale and CAT questionnaires were filled in by COPD patients. In addition, data about body mass index (BMI) and number of exacerbations in the previous year were collected, and BODCAT and BODEx were calculated. BODCAT is a multicomponent COPD index that consists of BMI, airflow obstruction, dyspnea and CAT score, while BODEx consists of BMI, airflow obstruction, dyspnea and history of exacerbations.

### Measurement of hematological and biochemical parameters

Blood samples from patients with stable COPD and from controls were collected between 7 and 9 a.m. by venipuncture of a large antecubital vein after overnight fasting, as recommended.

Three tubes were used for each participant as follows: a tube with K_3_-ethylenediaminetetraacetic acid (K_3_EDTA) anticoagulant (Greiner Bio-One, GmbH, Kremsmunster, Austria) was used for complete blood count (CBC) and IL-1β measurements, a tube with 3.2% sodium citrate anticoagulant (Becton, Dickinson and Company, Franklin Lakes, NJ, USA) was used for Fbg measurement and a serum tube with gel (Greiner Bio-One, GmbH, Kremsmunster, Austria) was used for CRP, creatinine (CREAT) and UA measurements. The venipuncture procedure and order of blood sampling and handling were performed according to recommendations for venous blood sampling [32]. CBC was performed within half an hour from blood sampling. Tubes with sodium citrate were centrifuged two times at 1500 x *g* for 15 min, as per national recommendations [33], and serum tubes with gel were centrifuged at 2000 *g* for 10 min, as recommended by the manufacturer. WBC counts were performed on a Sysmex XN-1000 analyzer (Sysmex Corporation, Kobe, Japan) as part of the CBC. The determination of leukocyte count is based on the flow cytometry method where laser light scattering technology is used. Fbg was measured using the optical method on a BCS XP analyzer (Siemens Healthcare Diagnostics, Marburg, Germany). Immunoturbidimetry was a method used for the CRP determination on a Cobas c501 analyzer (Roche Diagnostics GmbH, Mannheim, Germany). CREAT and UA concentrations were also measured on the Cobas c501 analyzer immediately after centrifugation procedure by using enzymatic colorimetric methods (Roche Diagnostics GmbH, Mannheim, Germany). Afterwards, the UCR ratio was calculated by dividing UA with CREAT values.

Internal quality control and external quality assessment were performed for measured parameters during the study period, according to HRN EN ISO 15189:2012 Medical laboratories—Requirements for quality and competence. The analyzer was calibrated according to the manufacturer’s instructions and checked by using commercial controls.

### Cytokine IL-1β determination

Concentrations of IL-1β in EDTA plasma samples obtained from patients with COPD and healthy individuals were measured using a ProcartaPlex High Sensitivity Assay, with a corresponding IL-1β bead set (Thermo Fisher Scientific, Waltman, MA, USA), according to the manufacturer’s recommendations. Briefly, 50 μL of antibody-coated magnetic beads were added per well into a 96-well plate and washed. Afterwards, 25 μL of samples or standards were added to a 25 μL universal assay buffer, and the plate was incubated for 30 min at room temperature (RT) and overnight at 4°C, with shaking. After the washing steps, 25 μL of detection antibodies were added to the wells and the plate was incubated for 30 min at RT, with shaking. After the washing, 50 μL of a streptavidin-phycoerythrin conjugate was added to the wells. After the incubation and washing steps, 50 μL of amplification reagent 1 was added to the wells, and the plate was incubated for 30 min at RT, with shaking. Then, amplification reagent 2 (50 μL) was added to the wells, and, following the incubation and washing steps, the beads were resuspended in 120 μL of reading buffer and analyzed by a Luminex 200 instrument (Luminex Corporation, Austin, TX, USA). The concentration of IL-1β was determined by interpolation from a standard curve using the xPONENT software package (Luminex Corporation, Austin, TX, USA).

### Spirometry

Spirometry was used as a method for diagnosing the airflow limitation. It was performed on a Master-Screen Pneumo spirometer (Jaeger, Germany), according to the recommendations of the European Respiratory Society and American Thoracic Society. The procedure was repeated at least three times, i.e. until two acceptable spirograms were obtained. The two largest FVC and FEV_1_ values had to show less than 5% variability, according to the standardized procedure [34]. The predicted values were the most commonly used European Community of Coal and Steel values [35]. Lung function parameters FEV_1_, FVC and FEV_1_/FVC were measured and statistically analyzed.

### Statistics

Kolmogorov-Smirnov test was used to assess the normality of distribution. All data were non-parametric, so they were presented as median with interquartile range, while only age was presented as median with minimum and maximum. Chi-squared test was used for comparison of males and females. Differences between controls and COPD patients were tested by Mann-Whitney Rank Sum test, while Kruskal-Wallis One Way Analysis of Variance on Rank test was used when comparing three or more groups of participants. Correlations were evaluated by Spearman Rank Order test and the obtained result were shown along with a correlation coefficient (r) and P value. Univariate and multivariate logistic regression analysis were also performed. Data were considered statistically significant if P < 0.05. Statistical analysis was performed by MedCalc statistical software, version 17.9.2. (MedCalc Software, Ostend, Belgium).

## Results

### UA and UCR levels are associated with inflammatory parameters in COPD patients

We assessed the lung function parameters and BMI as well as various inflammatory parameters (common, urate-related and cytokine IL-1β) in COPD patients in the stable phase of the disease and in healthy subjects matched with the patients by both age and sex (Table 1). Consistently with the inclusion and exclusion criteria, the spirometric data were lower in the COPD group (P<0.001), and the same goes for BMI (P = 0.012). UA and UCR levels were significantly increased in patients with COPD (P = 0.001 and P<0.001, respectively). Other inflammatory parameters, including IL-1β which might reflect inflammasome activation, were also significantly higher in the COPD group when compared to the control group (P<0.001).

**Table 1:** Baseline characteristics, lung function, hematological and biochemical parameters of healthy subjects and patients with stable COPD.

parameter	controls	COPD	P
	n = 95	n = 109	
age (years)	64	65	0.069
	(46–83)	(45–87)	
gender			0.121
males, n	49	69	
females, n	46	40	
BMI (kg/m^2^)	27.2	25.5	**0.012**
	(24.6–29.1)	(22.4–28.9)	
FEV_1_ (L)	2.60	1.08	**<0.001**
	(2.12–3.19)	(0.69–1.60)	
FEV_1_ (% pred.)	93	41	**<0.001**
	(86–104)	(28–62)	
FEV_1_/FVC	0.81	0.51	**<0.001**
	(0.77–0.88)	(0.41–0.59)	
WBC (x10^9^/L)	6.14	7.57	**<0.001**
	(5.15–7.42)	(6.56–8.95)	
CRP (mg/L)	1.47	2.34	**<0.001**
	(0.74–2.78)	(1.15–4.67)	
Fbg (g/L)	3.5	3.8	**<0.001**
	(3.1–3.8)	(3.4–4.5)	
CREAT (μmol/L)	79	72	0.062
	(67–88)	(60–84)	
UA (μmol/L)	292	333	**0.001**
	(249–348)	(289–381)	
UCR	3.88	4.59	**<0.001**
	(3.34–4.43)	(4.02–5.49)	
IL-1β (pg/mL)	0.105	6.902	**<0.001**
	(0.084–0.302)	(0.609–23.911)	

Age was presented as median (minimum to maximum), sex was presented as absolute number, while all other parameters were presented as median with interquartile range. Chi-squared test was used for comparison of males and females, while all other parameters were assessed by Mann-Whitney Rank Sum test. Data were considered significant if P<0.05.

BMI, body mass index; FEV_1_, forced expiratory volume in 1 second; FVC, forced vital capacity; WBC, white blood cells; CRP, C-reactive protein; Fbg, fibrinogen; CREAT, creatinine; UA, uric acid; UCR, uric acid to creatinine ratio; IL-1β, interleukin 1 beta.

We found no significant associations between lung function parameters, COPD-relevant scores or multicomponent indices and UA or UCR, while only UA was positively correlated with BMI (r = 0.425, P<0.001) in the COPD patients. However, both UA and UCR were significantly associated with all of the other inflammatory parameters, except for Fbg: WBC (r = 0.280, P = 0.001 for UA; r = 0.257, P = 0.007 for UCR), CRP (r = 0.324, P = 0.001 for UA; r = 0.303, P = 0.001 for UCR) and IL-1β (r = 0.337, P = 0.001 for UA; r = 0.282, P = 0.003 for UCR).

### Higher UCR levels are associated with COPD-related characteristics

Next, we subdivided COPD patients according to UA and UCR levels into low and high UA or UCR groups (Table 2). We found no statistically significant difference in UA and UCR values between the male and female patients probably because women were mostly in postmenopause, and therefore we used the same limit for both genders. The 95th percentile threshold of healthy controls was used as the cut-off value, i.e. 403 μmol/L for UA (≤403 μmol/L low and >403 μmol/L high) and 5.16 for UCR (≤5.16 low and >5.16 high). We explored possible differences in lung function, COPD-related scores and multicomponent indices, and hematological and biochemical parameters between low and high UA or UCR groups. The examined parameters had similar values in COPD patients with low and high UA concentrations in serum. Regarding UCR, no differences were found for common inflammatory parameters (WBC, CRP and Fbg) and dyspnea level (mMRC score). However, patients with higher UCR values exhibited worse other disease-related outcomes including lung function, symptoms, quality of life, history of exacerbations, and also had increased multicomponent indices (indicative of BMI, airflow obstruction, dyspnea, and CAT score or number of exacerbations in the previous year). In addition, the concentration of pro-inflammatory cytokine IL-1β was significantly elevated in COPD patients with higher UCR levels.

**Table 2:** Lung function, COPD-related scores and multicomponent indices, and hematological and biochemical parameters in COPD patients subdivided according to low and high UA and UCR levels.

parameter	low UA	high UA	P	low UCR	high UCR	P
	n = 83	n = 26		n = 72	n = 37	
FEV_1_ (L)	1.05	1.34	0.361	1.30	0.80	**0.001**
	(0.68–1.60)	(0.80–1.60)		(0.85–1.66)	(0.53–1.36)	
FEV_1_ (% pred.)	40	49	0.278	47	31	**0.006**
	(27–58)	(31–62)		(30–64)	(19–52)	
FEV_1_/FVC	0.50	0.53	0.527	0.54	0.47	**0.023**
	(0.40–0.59)	(0.46–0.59)		(0.42–0.61)	(0.40–0.54)	
mMRC score	2.00	1.00	0.780	1.50	2.00	0.231
	(1.00–2.75)	(1.00–3.00)		(1.00–2.00)	(1.00–3.00)	
CAT score	17.00	20.50	0.224	15.50	22.00	**0.001**
	(12.00–22.75)	(13.00–26.00)		(11.00–20.50)	(16.75–28.00)	
SGRQ-C score	44.60	48.65	0.536	41.10	49.40	**0.034**
	(27.13–63.05)	(32.00–64.60)		(26.20–60.50)	(35.95–75.63)	
previous exacerbations	1	1	0.938	1	1	**0.033**
	(0–2)	(0–2)		(0–1)	(0–2)	
BODCAT	5.00	4.00	0.867	4.00	6.00	**0.006**
	(3.00–7.00)	(3.00–7.00)		(2.00–7.00)	(4.00–8.00)	
BODEx	4.00	2.50	0.598	3.00	4.00	**0.049**
	(2.00–5.75)	(1.00–6.00)		(1.00–5.00)	(2.00–6.00)	
WBC (x10^9^/L)	7.40	7.67	0.359	7.57	7.61	0.675
	(6.35–8.86)	(6.75–9.17)		(6.47–8.86)	(6.61–9.26)	
CRP (mg/L)	2.19	4.20	0.193	2.15	3.32	0.150
	(1.15–4.55)	(1.10–5.90)		(1.12–4.38)	(1.20–6.52)	
Fbg (g/L)	3.7	3.9	0.235	3.8	3.9	0.411
	(3.4–4.4)	(3.6–4.5)		(3.4–4.4)	(3.5–4.7)	
IL-1β (pg/mL)	3.390	8.242	0.267	3.683	10.554	**0.033**
	(0.364–19.704)	(1.068–25.455)		(0.426–11.515)	(1.494–25.936)	

All of the parameters were assessed by Mann-Whitney Rank Sum test and are presented as median with interquartile range. Data were considered significant if P<0.05. Previous exacerbations were defined as the number of exacerbations in the previous year.

UA, uric acid; UCR, uric acid to creatinine ratio; FEV_1_, forced expiratory volume in 1 second; FVC, forced vital capacity; mMRC, modified Medical Research Council; CAT, COPD Assessment Test; SGRQ-C, St. George’s respiratory questionnaire for COPD patients; BODCAT–body mass index (BMI), airflow obstruction, dyspnea, score from COPD Assessment Test (CAT); BODEx–body mass index (BMI), airflow obstruction, dyspnea, previous exacerbations; WBC, white blood cells; CRP, C-reactive protein; Fbg, fibrinogen; IL-1β, interleukin 1 beta.

### Effect of disease severity on UA and UCR levels

**Fig 1:**
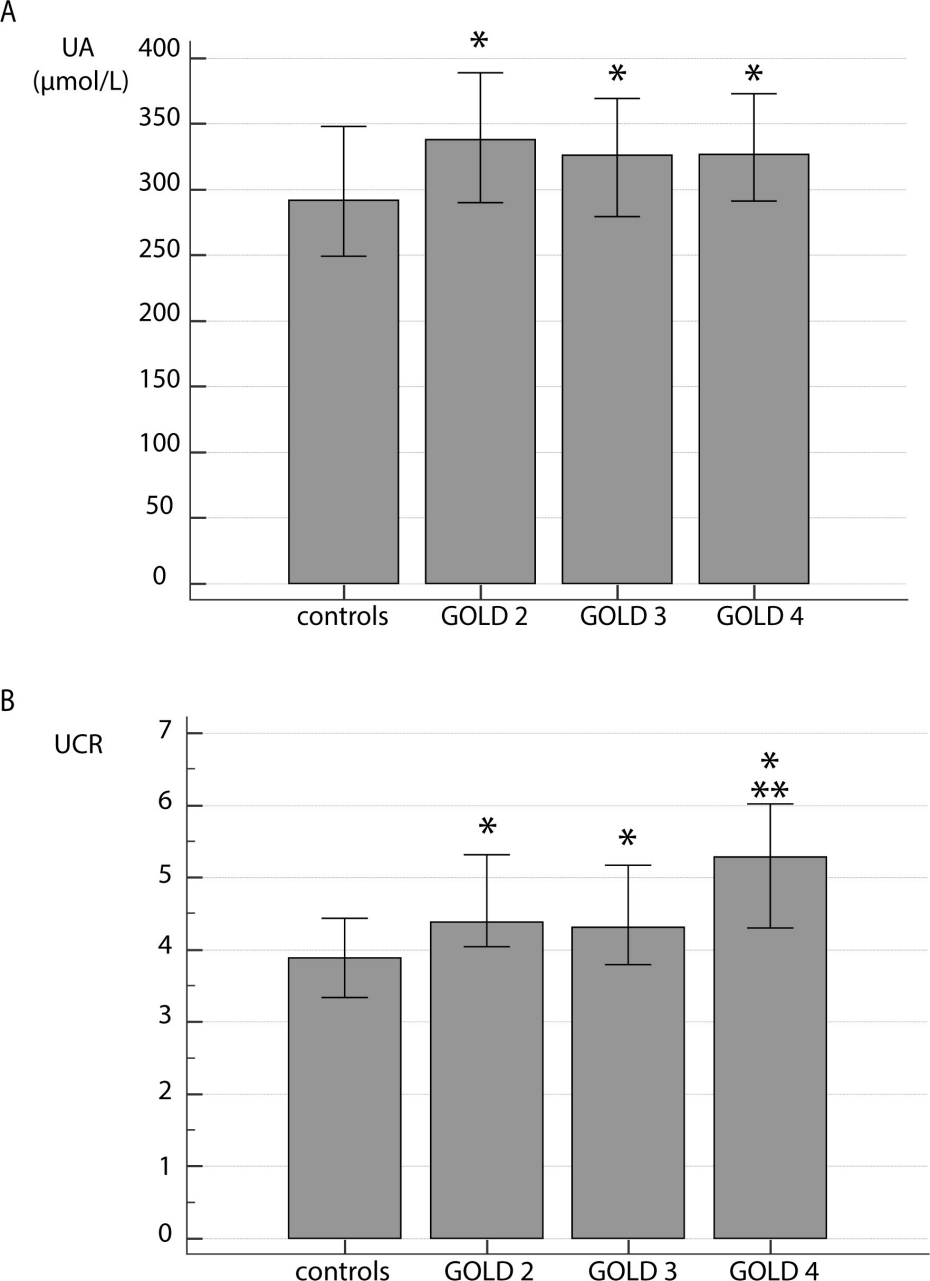
Influence of disease severity assessed by FEV_1_ on UA (A) and UCR (B) levels. COPD patients were subdivided by severity of airflow limitation into GOLD 2, GOLD 3 and GOLD 4 groups. Data are presented as median with interquartile range. Kruskal-Wallis test showed there was a significant difference between the groups (P = 0.007 for UA; P<0.001 for UCR), and *post-hoc* analysis was performed. * statistically significant difference in comparison to controls; ** statistically significant difference between GOLD 3 and GOLD 4.

We investigated the association of UA and UCR levels with the severity of airflow limitation, assessed by FEV_1_, in COPD patients subdivided in GOLD 2–4 stages (Fig 1). UA (Fig 1A) and UCR (Fig 1B) levels were higher in each disease grade compared to healthy subjects. It is important to note that both UA and UCR had already been increased in GOLD 2 classified patients, as in clinical practice this is often the lowest recognized disease stage. However, UA did not distinguish among GOLD stages, while UCR levels were elevated in GOLD 4 as compared to the GOLD 3 stage.

**Fig 2:**
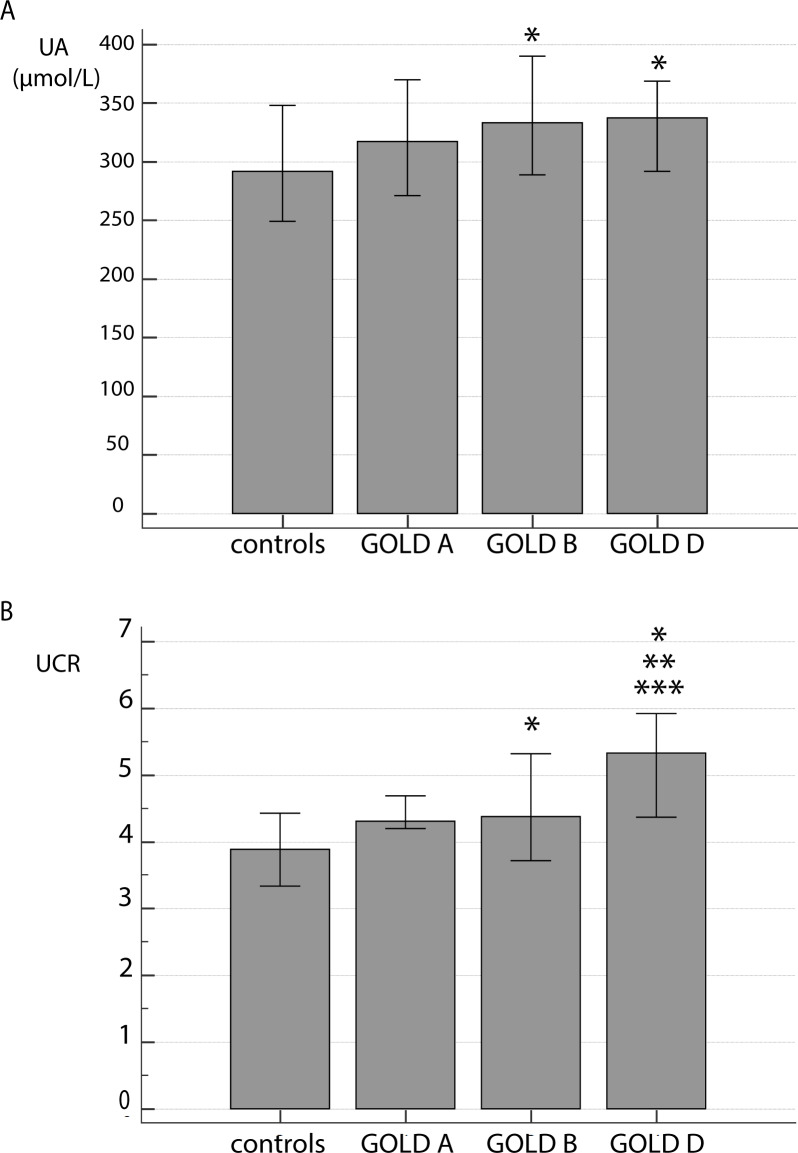
Influence of symptoms and history of exacerbations on UA (A) and UCR (B) levels. COPD patients were subdivided by severity of symptoms and exacerbations into GOLD A, GOLD B and GOLD D groups. Data are presented as median with interquartile range. Kruskal-Wallis test showed there was a significant difference between the groups (P = 0.007 for UA; P<0.001 for UCR), and *post-hoc* analysis was performed. * statistically significant difference in comparison to controls; ** statistically significant difference between GOLD A and GOLD D; *** statistically significant difference between GOLD B and GOLD D.

Next, we assessed UA and UCR levels in COPD patients subdivided into GOLD A–D groups according to their symptoms and history of exacerbations (Fig 2). UA (Fig 2A) and UCR (Fig 2B) values were increased in patients belonging to the GOLD B and GOLD D groups but were similar in the controls and GOLD A group. Only UCR was different among patients with various symptoms severity and frequency of exacerbations, as those in the GOLD D group had higher UCR levels than those in GOLD A or GOLD B groups.

### Effect of smoking on UA and UCR levels

Smoking is the main exogenous etiological factor in COPD, although only 15–20% smokers develop the disease. We subdivided COPD patients by smoking status into non-smokers, ex-smokers and smokers, while never and current smokers composed a group of healthy individuals (Table 3). The results showed that COPD non-smokers and COPD ex-smokers had higher levels of UA and UCR when compared to controls (both non-smokers and smokers). However, COPD current smokers had similar UA and UCR values as healthy subjects and significantly decreased values in comparison to COPD never and former smokers.

**Table 3:** Influence of smoking on UA and UCR levels.

smoking status	UA (μmol/L)	UCR
control non-smokers	307 (256–360)	4.01 (3.35–4.61)
control smokers	295 (240–337)	3.80 (3.31–4.35)
COPD non-smokers	329 (283–412) ^a^^,^^b^^,^^c^	4.85 (4.23–5.68) ^a^^,^^b^^,^^c^
COPD ex-smokers	351 (297–390) ^a^^,^^b^^,^^c^	5.00 (4.69–5.69) ^a^^,^^b^^,^^c^
COPD smokers	306 (275–338)	4.17 (3.74–4.69)
P	**0.001**	**<0.001**

Data are presented as median with interquartile range. Kruskal-Wallis test showed there was a significant difference between the groups, and *post-hoc* analysis was performed.

^a^ statistically significant difference in comparison to control non-smokers;

^b^ statistically significant difference in comparison to control smokers;

^c^ statistically significant difference in comparison to COPD smokers.

UA, uric acid; UCR, uric acid to creatinine ratio; COPD, chronic obstructive pulmonary disease.

### Levels of UA and UCR in COPD patients with different comorbidities and therapy regimes

We assessed the levels of UA and UCR in COPD patients with CVD or MS as the most common COPD comorbidities. Compared to patients without CVD (n = 53), COPD patients with CVD (n = 56) had significantly higher UA concentrations (313 (281–358) μmol/L *vs*. 352 (313–402) μmol/L, respectively; P = 0.008). In contrast, UCR did not differ between these COPD comorbidity subgroups (P = 0.957). Metabolic syndrome did not significantly affect UA (P = 0.225) and UCR (P = 0.297) levels among the COPD patients. One of the common parameters between CVD and MS is arterial hypertension (AH), and therefore we also assessed the UA and UCR levels in patients with AH in comparison with those without AH, but they were similar (P = 0.078 and 0.772, respectively).

Regarding common disease therapy, COPD patients were taking bronchodilators only or in combination with ICS, and they were subdivided according to their chronic inhalation therapy into four groups. No statistically significant differences in UA or UCR values were found between the treatment groups (S1 Table).

### Diagnostic characteristics of UA and UCR

Predictive values of explored inflammatory parameters were assessed by univariate logistic regression analysis (Table 4). Higher levels of all examined inflammatory parameters seems to be significant disease predictors, with IL-1β, Fbg and UCR being the most important, according to their odds ratios (OR) values.

**Table 4:** Univariate logistic regression analysis of inflammatory parameters.

parameter	OR	95% CI	P
WBC (x10^9^/L)	1.48	1.25–1.76	**<0.001**
CRP (mg/L)	1.24	1.09–1.41	**0.001**
Fbg (g/L)	2.55	1.65–3.95	**<0.001**
UA (μmol/L)	1.01	1.00–1.01	**0.002**
UCR	2.09	1.54–2.82	**<0.001**
IL-1β (pg/mL)	5.53	2.05–14.90	**0.001**

OR, odds ratio; CI, confidence interval; WBC, white blood cells; CRP, C-reactive protein; Fbg, fibrinogen; UA, uric acid; UCR, uric acid to creatinine ratio; IL-1β, interleukin 1 beta.

Next, we wanted to establish multiparameter models that include UA or UCR and that might become useful in distinguishing between healthy and COPD individuals. We wanted to offer the best model with only routinely measured parameters in the laboratory as well as a model that would include the pro-inflammatory cytokine IL-1β whose OR value was the highest among all individual parameters. We analyzed possible combinations with multivariate logistic regression analysis. The results for UA with (Model 1) or without (Model 2) IL-1β, as this cytokine is not a part of everyday laboratory practice, are shown in Table 5, and those for UCR in Table 6. For UA, Model 2 correctly classified 67% of cases, while Model 1 correctly classified 86% of cases with an AUC of 0.952. For UCR, the results were even better, and Model 1 correctly classified 90% of cases with an AUC of 0.964, while Model 2 correctly classified 74% of the cases.

**Table 5:** Inflammatory multiparameter models with UA assessed by multivariate logistic regression analysis.

Model 1				Model 2			
parameter	OR	95% CI	P	parameter	OR	95% CI	P
WBC (x10^9^/L)	1.20	0.97–1.50	0.095	WBC (x10^9^/L)	1.30	1.10–1.54	**0.002**
Fbg (g/L)	2.01	0.98–4.10	0.056	CRP (mg/L)	1.08	0.95–1.23	0.236
UA (μmol/L)	1.01	1.00–1.02	**0.001**	Fbg (g/L)	1.82	1.08–3.07	**0.025**
IL-1β (pg/mL)	5.10	2.15–12.09	**0.001**	UA (μmol/L)	1.00	1.00–1.01	**0.022**
The analysis gave results with 86% of correctly classified cases and AUC of 0.952 (0.913–0.977).				The analysis gave results with 67% of correctly classified cases and AUC of 0.753 (0.688–0.810).			

OR, odds ratio; CI, confidence interval; WBC, white blood cells; Fbg, fibrinogen; CRP, C-reactive protein; UA, uric acid; IL-1β, interleukin 1 beta; AUC, area under the curve.

**Table 6:** Inflammatory multiparameter models with UCR assessed by multivariate logistic regression analysis.

Model 1				Model 2			
parameter	OR	95% CI	P	parameter	OR	95% CI	P
WBC (x10^9^/L)	1.22	0.96–1.56	0.112	WBC (x10^9^/L)	1.29	1.08–1.55	**0.005**
CRP (mg/L)	1.18	0.99–1.41	0.063	CRP (mg/L)	1.12	0.98–1.26	0.087
UCR	3.17	1.87–5.38	**<0.001**	Fbg (g/L)	1.56	0.91–2.69	0.108
IL-1β (pg/mL)	5.11	2.33–11.20	**<0.001**	UCR	1.97	1.42–2.72	**<0.001**
The analysis gave results with 90% of correctly classified cases and AUC of 0.964 (0.928–0.985).				The analysis gave results with 74% of correctly classified cases and AUC of 0.791 (0.729–0.845).			

OR, odds ratio; CI, confidence interval; WBC, white blood cells; CRP, C-reactive protein; UCR, uric acid to creatinine ratio; Fbg, fibrinogen; IL-1β, interleukin 1 beta; AUC, area under the curve.

## Discussion

This study found higher levels of UA and its corrective ratio with creatinine in the peripheral blood of patients with COPD when compared to age- and sex-matched healthy subjects. General inflammatory markers (WBC, CRP, Fbg) and cytokine IL-1β were also increased in COPD patients and correlation analysis showed that they were all significantly associated with both UA and UCR, except for Fbg. On the other hand, we found no significant associations between lung function parameters, COPD-relevant scores or multicomponent indices and UA or UCR. However, when the patients were subdivided according to low and high UA or UCR levels, decreased spirometric values and increased number of exacerbations in previous years, CAT and SGRQ-C scores, BODCAT and BODEx indices as well as IL-1β concentration were present only in those with higher UCR levels, while no difference was found with an applied cut-off value for UA.

Higher UA [6,7,36] or UCR [7] levels in COPD patients in comparison to healthy individuals were observed by other studies, although some demonstrated no differences [37,38] or even lower levels [39]. Conflicting results regarding the association of UA with lung function were also reported, and negative [5,40–43], positive [44] or no association [40] were found, and this depended mostly on the sample size and method used (observational and Mendelian randomization analysis). Only a few studies explored the UA association with other determinants of worse COPD outcomes, including physical capacity [41], dyspnea [43], acute exacerbation of COPD [41,42] and mortality [42,45]. Regarding UCR, Durmus Kocak et al. demonstrated a positive correlation of UCR, but not of UA, with the CAT score [7]. They also demonstrated that UCR can be more useful than UA in predicting COPD severity and exacerbation risk, especially at higher cut-off values [7]. Garcia-Pachon et al. showed that COPD patients with increased UCR had lower FEV_1_ (% pred.) and FVC (% pred.) values and a higher level of dyspnea assessed by mMRC score compared to those with decreased UCR, while only FVC (% pred.) differed when COPD patients were subdivided according to higher and lower concentrations of UA [43]. In addition, the aforementioned authors demonstrated no significant correlations between serum UA and any of the functional and clinical parameters examined, and contrary to this, UCR negatively correlated with spirometric values and positively with dyspnea severity [43].

Several studies assessed the severity of airflow limitation and its association with UA levels. Sarangi et al. observed a trend indicating higher concentrations of UA in GOLD 4 stage compared to other stages, but it was statistically insignificant [36]. In COPD patients admitted for acute exacerbation, UA admission levels were higher in patients with more severe airflow limitation, i.e. higher in those with GOLD 3 and GOLD 4 than in those with GOLD 1 and GOLD 2 stages [42]. Similarly, in a large epidemiological study, subjects with moderate to severe airflow limitation had higher concentrations of serum UA than subjects with mild airflow limitation as well as those without airflow limitation [46]. Recently, Dishan et al. found differences in the UA and UCR levels associated with severity of airflow obstruction [47]. Our results showed that in COPD patients, both UA and UCR were higher in all examined GOLD stages when compared to controls, even in GOLD 2 that is in clinical practice usually the lowest grade when individuals seek medical help. No differences among stages were found for UA. Contrary to this, UCR distinguished the GOLD 3 from GOLD 4 grade. When patients with COPD were subdivided according to their symptoms and history of exacerbations (ABCD assessment), increased UA and UCR were determined in GOLD B and GOLD D groups (groups with worse symptoms), with no significant difference between the patients in GOLD A group and healthy subjects. Once again, only UCR could differentiate among the GOLD groups (GOLD A and GOLD D as well as GOLD B and GOLD D). To the best of our knowledge, levels of UA have thus far not been associated with GOLD A–D groups of COPD patients, while UCR has not been associated with either airflow limitation or symptoms/exacerbations severity.

Regarding smoking, the COPD smokers in our study had decreased UA and UCR levels compared to COPD never and former smokers and similar to those of controls. This is in accordance with the results of Sarangi et al., who observed higher UA concentrations in COPD non-smokers than in COPD smokers, although their results were not statistically significant [36]. In studies with healthy individuals, current smokers also had significantly lower UA levels compared to non-smokers and/or ex-smokers [48–51], with only a few rare exceptions [52]. It was suggested that decreased UA concentrations in smokers could be attributed to its depletion and/or reduced endogenous production as a result of chronic exposure to cigarette smoke that is a significant source of oxidant molecules, including oxygen free radicals [51]. A decrease of UA in smokers could also be explained by XO inactivation with tobacco smoke compound cyanide [53]. However, it was shown that XO activity was increased in the epithelial lining fluid of COPD patients [54], as well as in the induced sputum of COPD patients [55]. Although higher XO activity in COPD airways does not necessarily reflect its circulating levels in peripheral blood, as XO is a rate-limiting enzyme of purine catabolism with UA being the final product in humans, the scenario involving an increased XO is more likely to be applied in our context with elevated UA (and UCR) levels present in patients’ sera. It has been reported that hypoxia can upregulate XO gene expression and activity [56] and hypoxia is associated with COPD pathophysiology. During its reaction, XO produces reactive oxygen species, and increased antioxidants, such as UA, could be a part of the adaptive mechanism to oxidative stress. In addition, patients with CVD had higher UA concentrations than those without CVD [42], which is in accordance with our study and might also be a compensatory response designed to counteract excessive oxidative stress.

Finally, in this study we explored the diagnostic characteristics of UA and UCR and found a great predictive potential in COPD diagnosis for UCR and IL-1β as individual parameters. We also proposed two models that included UA or UCR and several common inflammatory parameters with or without IL-1β, as this cytokine is not a part of everyday laboratory practice and for now is too expensive to be routinely measured. While in models without IL-1β only a combination with UCR gave satisfactory results that correctly classified 74% of cases with an AUC close to 0.8, when this pro-inflammatory cytokine was included in combinations with UA as well as with UCR, excellent results were obtained and such models could correctly classify even 86% and 90% of cases, respectively. These data might suggest, although indirectly, an association between UA and IL-1β, probably at least partly through UA-induced inflammasome activation. Certainly, further studies are needed to explore this assumption.

Although we have presented some novel and interesting results, our study does possess some limitations. It did not include COPD patients from the GOLD C group or the GOLD 1 stage and it would be useful to assess UA and UCR levels in this earliest stage of the disease and clarify if those parameters could differ from a healthy population even at the beginning of disease development. However, it is well-known in clinical practice that the GOLD 1 group of COPD patients rarely contact their physician due to very mild symptoms, while the GOLD C category do not have many symptoms and are not usually frequent exacerbators. A larger number of participants should be recruited for further studies and a longitudinal study design should be considered.

In conclusion, this study has shown that both UA and UCR levels were higher in COPD patients compared to healthy subjects and were associated with common inflammatory parameters as well as IL-1β. However, higher values of UCR only were associated with lung function, history of exacerbations, COPD-related scores and multicomponent indices. In contrast to UA, UCR distinguished between some disease severity grades according to both airflow limitation as well as symptoms and exacerbations. Also, UCR seems to be a better COPD predictor than UA and multiparameter inflammatory models with UCR showed better diagnostic characteristics, considering that it was a simple model with only routine laboratory parameters (WBC, CRP, Fbg) and included the IL-1β cytokine which correctly classified 74% and 90% of cases, respectively. We suggest that UA might be a useful biomarker when combined with IL-1β, while UCR might be even more informative and useful in overall COPD assessments.

## Supporting information

S1 Table Influence of common COPD therapy on UA and UCR levels. COPD patients were subdivided according to their therapy regimes as follows: COPD patients in therapy 1 group received monotherapy of long-acting bronchodilator (LABAs or LAMAs) with or without short-acting bronchodilator (SABAs or SAMAs), in therapy 2 group received dual long-acting bronchodilators LABA and LAMA, in therapy 3 group received combination of long-acting bronchodilator with ICS, and in therapy 4 group received triple therapy with added LAMA. Each patient belongs to only one therapy group. UA, uric acid; UCR, uric acid to creatinine ratio; LABA, long-acting β_2_-agonist; LAMA, long-acting muscarinic antagonist; SABA, short-acting β_2_-agonist; SAMA, short-acting muscarinic antagonist; ICS, inhaled corticosteroids.
(DOCX)
