# Supplementary material for: Uric acid and uric acid to creatinine ratio in the assessment of chronic obstructive pulmonary disease: Potential biomarkers in multicomponent models comprising IL-1beta
Source: PLoS One. 2020 Jun 5;15(6):e0234363. doi: 10.1371/journal.pone.0234363 (PMC7274385; doi:10.1371/journal.pone.0234363)
Supplement: S1 Table — COPD patients were subdivided according to their therapy regimes as follows: COPD patients in therapy 1 group received monotherapy of long-acting bronchodilator (LABAs or LAMAs) with or without short-acting bronchodilator (SABAs or SAMAs), in therapy 2 group received dual long-acting bronchodilators LABA and LAMA, in therapy 3 group received combination of long-acting bronchodilator with ICS, and in therapy 4 group received triple therapy with added LAMA. Each patient belongs to only one therapy group. UA, uric acid; UCR, uric acid to creatinine ratio; LABA, long-acting β2-agonist; LAMA, long-acting muscarinic antagonist; SABA, short-acting β2-agonist; SAMA, short-acting muscarinic antagonist; ICS, inhaled corticosteroids. (DOCX) [file pone.0234363.s001.docx]

**S1 Table. Influence of common COPD therapy on UA and UCR levels.**

|  | UA (µmol/L) | P | UCR | P |
| --- | --- | --- | --- | --- |
| COPD on therapy 1 (n = 20) | 308 (257 - 362) | 0.611 | 4.31 (3.83 - 4.89) | 0.857 |
| COPD without therapy 1 (n = 89) | 320 (259 - 388) |  | 4.32 (3.63 - 5.28) |  |
| COPD on therapy 2 (n = 32) | 339 (265 - 426) | 0.240 | 4.38 (3.39 - 4.94) | 0.532 |
| COPD without therapy 2 (n = 77) | 313 (250 - 363) |  | 4.30 (3.76 - 5.30) |  |
| COPD on therapy 3 (n = 20) | 334 (216 - 363) | 0.691 | 4.69 (3.98 - 5.62) | 0.262 |
| COPD without therapy 3 (n = 89) | 315 (261 - 388) |  | 4.30 (3.63 - 5.00) |  |
| COPD on therapy 4 (n = 37) | 313 (255 - 378) | 0.591 | 4.18 (3.59 - 5.14) | 0.636 |
| COPD without therapy 4 (n = 72) | 328 (263 - 375) |  | 4.44 (3.77 - 5.10) |  |

COPD patients were subdivided according to their therapy regimes as follows: COPD patients in therapy 1 group received monotherapy of long-acting bronchodilator (LABAs or LAMAs) with or without short-acting bronchodilator (SABAs or SAMAs), in therapy 2 group received dual long-acting bronchodilators LABA and LAMA, in therapy 3 group received combination of long-acting bronchodilator with ICS, and in therapy 4 group received triple therapy with added LAMA. Each patient belongs to only one therapy group.

UA, uric acid; UCR, uric acid to creatinine ratio; LABA, long-acting β_2_-agonist; LAMA, long-acting muscarinic antagonist; SABA, short-acting β_2_-agonist; SAMA, short-acting muscarinic antagonist; ICS, inhaled corticosteroids.
